# World Health Organization bacterial priority pathogens in skin and soft tissue infections: prevalence, antimicrobial resistance, and risk factors in Mwanza, Tanzania

**DOI:** 10.1016/j.ijregi.2026.100926

**Published:** 2026-05-25

**Authors:** Vitus Silago, Katarina Oravcova, Louise Matthews, Stephen E. Mshana, Heike Claus, Jeremiah Seni

**Affiliations:** 1 Catholic University of Health and Allied Sciences, Weill Bugando School of Medicine, Department of Microbiology and Immunology, Mwanza, Tanzania; 2 University of Würzburg, Institute for Hygiene and Microbiology, Würzburg, Germany; 3 University of Glasgow, School of Biodiversity, One Health & Veterinary Medicine, Glasgow, UK

**Keywords:** WHO bacterial priority pathogens, Action plan on antimicrobial resistance, Skin and soft tissue infections, Gram-negative bacteria, Gram-positive bacteria

## Abstract

**Objectives:**

Antimicrobial resistance, particularly among World Health Organization bacterial priority pathogens (WHO BPPs), poses a major global health threat. We assessed the epidemiology of wound infections, including bacterial profiles, WHO BPP burden, and associated factors, during and after the implementation of the National Action Plan on Antimicrobial Resistance (NAP-AMR) in Mwanza, Tanzania.

**Methods:**

A cross-sectional study was conducted during (June 2019 to June 2020) and after (March to August 2023) NAP-AMR. Pus samples were cultured aerobically. Isolates were identified using Vitek mass spectrometry and 16S ribosomal ribonucleic acid sequencing. Antimicrobial susceptibility testing and minimum inhibitory concentrations were determined using Vitek 2.

**Results:**

Among 630 patients (median age 29 years; interquartile range 15-47), 257 (40.8%) had positive cultures. Skin and soft tissue infections increased significantly after NAP-AMR (27.8% vs 68.3%, *P* < 0.001). Resistance to third-generation cephalosporins (3GCs) increased significantly in *Escherichia coli (P* < 0.05). Additionally, meropenem resistance among Gram-negative bacteria increased from 2.6% to 21.0% (*P* = 0.327), primarily attributable to the increased isolation of *Acinetobacter* spp. and *Pseudomonas aeruginosa* after NAP-AMR. WHO BPPs accounted for 45.2% of isolates, and extended-spectrum beta-lactamase-producing Enterobacterales increased significantly (64.8% vs 82.5%, *P* = 0.025), particularly in higher-tier hospitals (45.4% vs 77.9%, *P* = 0.002).

**Conclusion:**

After NAP-AMR, we recorded significant increases in resistance to 3GCs among *E. coli*, increased resistance to meropenem among Gram-negative bacteria (notably *Acinetobacter* spp. and *P. aeruginosa*), and a high proportion of WHO BPPs, particularly extended-spectrum beta-lactamase-producing Enterobacterales, potentially reflecting improved diagnostics, enhanced laboratory capacity, or heightened antimicrobial use during the coronavirus disease 2019 pandemic. These findings underscore the need for sustained surveillance and strengthened antimicrobial stewardship.

## Highlights

• Resistance of *Escherichia coli* to third-generation cephalosporins increased significantly after National Action Plan on Antimicrobial Resistance (AMR), indicating escalating therapeutic challenges.
• Nearly half of all isolates were classified as World Health Organization bacterial priority pathogens, highlighting the substantial clinical and public health burden of AMR in skin and soft tissue infections.
• Meropenem resistance among Gram-negative bacteria increased from 2.6% to 21.0% (*P* = 0.327), largely driven by increased isolation of *Acinetobacter* spp. and *Pseudomonas aeruginosa* after National Action Plan on AMR implementation.
• Enhanced bacteriology laboratory capacity for culture and antimicrobial susceptibility testing strengthened antimicrobial stewardship and may be associated with a slight decline in AMR among *Klebsiella* spp.

## Introduction

Antimicrobial resistance (AMR) has emerged as a major global public health threat, with infections caused by drug-resistant bacteria posing increasing challenges to effective clinical management [1]. The World Health Organization (WHO) has identified a set of “bacterial priority pathogens” (BPPs) that are particularly concerning owing to their high and rising resistance to commonly used antibiotics [2,3]. Among these, the critical group comprises third-generation cephalosporin (3GC)–resistant Enterobacterales, including extended-spectrum beta-lactamase-producing Enterobacterales (ESBL-PE), and carbapenem-resistant Enterobacterales and *Acinetobacter baumannii*. The high-priority group includes carbapenem-resistant *Pseudomonas aeruginosa* and methicillin-resistant *Staphylococcus aureus* (MRSA) [2,3]. These pathogens are associated with increased morbidity and mortality and complicate the management of infections such as skin and soft tissue infections (SSTIs) [1].

SSTIs are common clinical conditions caused by a variety of bacterial pathogens, some of which exhibit multidrug resistance, making effective treatment increasingly difficult [4]. Resistance phenotypes such as MRSA and ESBL-PE have been reported widely in health care settings, particularly in low- and middle-income countries, including Tanzania [5]. The rise of these resistant pathogens underscores the urgent need for timely and effective antibiotic treatment in SSTIs to prevent complications.

In response to the global AMR threat, WHO launched the Global Action Plan on Antimicrobial Resistance in 2016, urging countries to develop national strategies to mitigate resistance [6]. In Tanzania, the Ministry of Health operationalized this strategy through the National Action Plan on Antimicrobial Resistance (NAP-AMR) 2017-2022 [7]. The NAP-AMR outlined five key objectives: raising AMR awareness, improving surveillance and research, reducing infections through sanitation and infection prevention, optimizing antimicrobial use in human and animal health, and promoting investment in new medicines, diagnostics, and vaccines [7]. Initially, interventions aimed at improving AMR awareness, promoting rational antibiotic use, and strengthening infection prevention and control (IPC) were primarily concentrated in selected zonal and regional referral hospitals, with no engagement of district hospitals despite their pivotal role in primary health care delivery. Furthermore, district hospitals lacked microbiology laboratory capacity for bacterial culture and antimicrobial susceptibility testing (AST).

To bridge these gaps, the Supporting the National Action Plan on Antimicrobial Resistance (SNAP-AMR) project was implemented from June 2019 to June 2020, expanding NAP-AMR activities to three district hospitals in Mwanza: Sumve District Designated Hospital (SDDH), Magu District Hospital (MDH), and Misungwi District Hospital (MisDH). In addition, the project established bacteriology laboratory capacity within these facilities to support bacterial culture, AST, and the detection of key resistance phenotypes, including ESBL-PE, carbapenem-resistant Gram-negative bacteria (CR-GNB), and MRSA.

Therefore, this study aimed to determine the epidemiology of wound infections (bacterial profiles, proportion of WHO BPPs, and factors associated with infections caused by WHO BPPs) among patients attending district, regional, and zonal referral hospitals in Mwanza, Tanzania, during and after NAP-AMR implementation. The findings provide critical insights to guide future strategies for mitigating the growing burden of AMR in the region.

## Methods

### *Study design*

This hospital-based cross-sectional study was conducted during the NAP-AMR period (from June 2019 to June 2020) and after NAP-AMR (from March to July 2023) among patients with a clinical diagnosis of SSTIs attending or admitted to three district hospitals, a regional referral hospital, and a zonal referral hospital in Tanzania. Bugando Medical Centre (BMC), with approximately 1000 beds, serves as a zonal referral hospital for seven Lake Zone regions (Mwanza, Simiyu, Geita, Kagera, Kigoma, Shinyanga, and Mara), covering an estimated population of 18.8 million [8,9]. Sekou Toure Regional Referral Hospital (SRRH) has approximately 450 beds and serves the Mwanza region with a population of approximately 3.6 million [9]. MDH, SDDH, and MisDH have approximately 150, 265, and 128 beds, respectively, serving the districts of Magu, Kwimba, and Misungwi, with a combined population of 1.3 million [9]. BMC and Sekou Toure Regional Referral Hospital are classified as “higher-tier hospitals,” while MDH, SDDH, and MisDH are classified as “lower-tier hospitals.”

### *Interventions during and after NAP-AMR*

The interventions implemented during the NAP-AMR period primarily focused on education aimed at increasing AMR awareness and promoting rational antibiotic use among health care providers and the community, strengthening IPC practices, and improving diagnostic stewardship and laboratory procedures. These interventions were further expanded through the SNAP-AMR project between June 2019 and June 2020 to three district hospitals that had not originally been part of the routine NAP-AMR implementation. In addition, the SNAP-AMR project established bacteriology laboratory capacity in these district hospitals. Following the conclusion of the NAP-AMR period, findings from the first cohort were presented to the respective hospitals. During the second cohort, after NAP-AMR, interventions were limited to education promoting rational antimicrobial prescribing, training on the utilization of bacteriology laboratories, and refresher training on diagnostic stewardship and laboratory procedures.

### *Patient selection, enrollment, and sample and data collection*

Patients were enrolled without duplication in their respective wards of admission or clinics of attendance in each hospital by the attending doctors, who made a clinical diagnosis of SSTI based on their assessment of the patient’s signs and symptoms [10]. Pus in sterile specimen containers or pus swabs in Stuart transport medium (HiMedia Laboratories Pvt. Ltd., Mumbai, India) were collected and sent to the laboratory of the respective hospital for culture and AST. Once the laboratory received a sample, the patient was eligible for enrollment in this study. A research assistant was then notified and provided with the patient’s details (name, hospital registration number, and ward/clinic information) for tracking, obtaining voluntary consent, and collecting additional data, including current antibiotic use and history of hospital admission or antibiotic use, using a structured questionnaire.

### *Laboratory procedures*

#### Bacteriological culture, confirmation of isolates, and AST

Pus and pus swab samples were inoculated and streaked onto 5% sheep blood agar and MacConkey agar plates, followed by aerobic incubation at 35 ± 2°C for 24 hours. After incubation, significant microbes from positive cultures were identified to the genus and/or species level using in-house biochemical tests [11], followed by AST using the Kirby-Bauer disk diffusion method [12]. To confirm the etiological role of rare or uncommon bacterial species in wound infection, a second pus/pus swab sample was collected within three days from patients whose initial culture demonstrated pure and significant growth extending to the third quadrant, and inoculated onto sheep blood agar and MacConkey agar plates. The zones of inhibition around antimicrobial discs were measured in millimeters and interpreted as resistant, intermediate, or susceptible according to the Clinical & Laboratory Standards Institute guidelines (2019 [13] and 2020 [14] during NAP-AMR and 2023 [15] after NAP-AMR) to guide the rational management of patients. All procedures were performed in the respective hospital laboratories following standardized protocols for reproducibility. Data were collected from each hospital and stored at the Microbiology Laboratory of the Catholic University of Health and Allied Sciences (https://www.bugando.ac.tz/schools/school_of_medicine/departments/microbiology/index.php). Bacterial isolates were stored in 20% glycerol-supplemented brain heart infusion broth and archived at -80°C.

Given the limited discriminatory capacity of conventional biochemical identification methods for accurate species-level characterization of bacterial isolates, all bacterial isolates were further validated using automated analyzers between August and October 2023 at the Institute for Hygiene and Microbiology, Würzburg, Germany. Species identification was performed using matrix-assisted laser desorption/ionization time-of-flight mass spectrometry on a VITEK MS^TM^ (bioMérieux, Nürtingen, Germany), with 16S ribosomal ribonucleic acid Sanger sequencing [16] used in cases with uncertain results. The Vitek 2 system (bioMérieux, Nürtingen, Germany) was used for AST. The following AST cards were used: AST-P654 for *Staphylococcus* spp. and other Gram-positive bacteria (GPB); AST-N214 for Enterobacterales and *Acinetobacter* spp.; AST-P655 for *Enterococcus* spp. and *Streptococcus* spp.; and AST-N248 for *Pseudomonas* spp. All AST cards were obtained from bioMérieux. The minimum inhibitory concentrations were interpreted according to the European Committee on AST clinical breakpoints, version 13.0 (2023) [17]. The WHO BPPs in the current study included MRSA, ESBL-PE (*Escherichia coli* and *Klebsiella pneumoniae*), and CR-GNB (*A. baumannii, P. aeruginosa*, and Enterobacterales). The WHO BPPs in this study were determined using minimum inhibitory concentrations from the Vitek 2 system interpreted by European Committee on AST version 13.0 [17] and the WHO BPP list of 2017 [2]. In this study, intermediate AST results were interpreted as resistant. The final results used for analysis were based on results from automated analyzers (Vitek MS^TM^ and Vitek 2) obtained in Germany.

### *Quality control*

The BMC laboratory was accredited by Kenya Accreditation Service, while all other laboratories were enrolled in and actively participated in external quality assessment programs provided by the National Public Health Laboratory of Tanzania. All culture media were prepared in accordance with the manufacturer’s instructions, and underwent sterility and performance verification before use. In addition, AST discs underwent quality control testing with every new batch and on a weekly basis to ensure optimal performance across all testing laboratories. *E. coli* American Type Culture Collection 25922 and *S. aureus* American Type Culture Collection 25923 were used as the control microorganisms in both settings in Tanzania and Germany.

### *Data management and statistical analysis*

The collected data were cleaned and coded in Microsoft Excel before being transferred to Stata version 15.0 (StataCorp LLC, College Station, TX, USA) for analysis. Categorical variables were examined using tabulation and the results were reported as percentages and proportions. Descriptive statistics were used to assess the central tendency and variability, and the findings were presented as median and interquartile range (IQR). Chi-square and two-sample proportion tests were used to compare independent categorical variables, while the Wilcoxon rank-sum test was used to compare the medians of independent continuous variables. A logistic regression model was used to assess the association between SSTIs caused by WHO BPPs and the categorical independent variables. Statistical significance was defined as *P* < 0.05 with a 95% confidence interval (CI). Independent variables with *P* < 0.05 were subjected to multivariable regression analysis. These predictors were subsequently included in a multivariable regression to adjust for potential confounders and determine the independent predictors of SSTIs caused by WHO BPPs.

## Results

### *Sociodemographic and clinical characteristics of patients clinically diagnosed with SSTIs*

A total of 630 patients with clinically diagnosed SSTIs were enrolled, including 428 during NAP-AMR and 202 afterward. The median age was 29 years (IQR 15-47), and 54.8% (345/630) were female. Overall, 59.7% (376/630) were recruited from higher-tier hospitals. During NAP-AMR, a larger proportion enrolled from lower-tier hospitals (53.0% vs 13.4%, *P* < 0.001), whereas after NAP-AMR, most participants were from higher-tier hospitals (86.6% vs 47.0%, *P* < 0.001). Inpatients accounted for 65.3% (411/630), with higher proportions after NAP-AMR (72.3% vs 61.9%, *P* = 0.017). Patients were primarily enrolled from medical (43.6%, 275/630), surgical (25.4%, 160/630), and pediatric (14.3%, 90/630) wards, with recruitment from surgical wards increasing after NAP-AMR (39.6% vs 18.7%, *P* = 0.002). At enrollment, 43.1% (271/630) of patients were receiving antibacterial therapy, a proportion significantly higher during NAP-AMR than after (62.9% vs 1.0%, *P* = 0.034); 41.7% (263/630) had experienced fever in the three months before enrollment, more commonly during NAP-AMR than after (47.0% vs 30.7%, *P* = 0.012) (Table 1).

**Table 1:** Sociodemographic and clinical characteristics of patients clinically diagnosed with SSTIs.

Characteristics	Categories	All (n = 630)	During NAP-AMR (n = 428)	After NAP-AMR (n = 202)	*P*-value^a^
		n (%)	n (%)	n (%)	
Median [IQR] age, years		29 [15-47]	27 [15-44]	31 [15-49]	0.337
Sex	Male	285 (45.2)	182 (42.5)	103 (51.9)	0.063
	Female	345 (54.8)	246 (57.5)	99 (49.0)	0.075
Residency	Rural	352 (55.9)	241 (56.3)	111 (54.9)	0.403
	Urban	278 (44.1)	187 (43.7)	91 (45.1)	0.413
Education level	None	183 (29.0)	136 (31.8)	47 (23.3)	0.135
	Primary	277 (43.9)	200 (46.7)	77 (38.1)	0.098
	Secondary	127 (20.1)	72 (16.8)	55 (27.2)	0.078
	Tertiary	43 (7.0)	20 (4.7)	23 (11.4)	0.213
Occupation	None	242 (38.4)	165 (38.6)	77 (38.1)	0.470
	Employed	53 (8.4)	27 (6.3)	26 (12.9)	0.207
	Self-employed	335 (53.2)	236 (55.1)	99 (49.0)	0.153
Level of health care facility	Lower-tier	254 (40.3)	227 (53.0)	27 (13.4)	**<0.001**
	Higher-tier	376 (59.7)	201 (47.0)	175 (86.6)	**<0.001**
Patient category	Outpatient	219 (34.7)	163 (38.1)	56 (27.7)	0.080
	Inpatient	411 (65.3)	265 (61.9)	146 (72.3)	**0.017**
Ward or clinic of enrollment	Medical	275 (43.6)	198 (46.3)	77 (38.1)	0.109
	Surgical	160 (25.4)	80 (18.7)	80 (39.6)	**0.002**
	Pediatric	90 (14.3)	85 (19.9)	5 (2.5)	0.167
	Others^b^	105 (16.6)	65 (15.1)	40 (19.8)	0.266
Receiving antibacterials during enrollment	Yes	271 (43.1)	269 (62.9)	2 (1.0)	**0.034**
	No	359 (56.9)	159 (37.1)	200 (99.0)	**<0.001**
Types of antibacterials in use during enrollment	Penicillins	131 (48.3)	130 (48.3)	1 (50.0)	0.513
	Cephalosporins	89 (32.8)	88 (32.7)	1 (50.0)	0.643
	Ciprofloxacin	67 (24.7)	67 (24.9)	0 (0.0)	NA
	Metronidazole	57 (21.0)	57 (21.2)	0 (0.0)	NA
	Others^c^	46 (16.9)	45 (16.7)	1 (50.0)	0.807
History of fever in past 3 months	Yes	263 (41.7)	201 (47.0)	62 (30.7)	**0.012**
	No	367 (58.3)	227 (53.0)	140 (69.3)	**<0.001**
History of admission in past 3 months	Yes	111 (17.6)	70 (16.4)	41 (20.3)	0.302
	No	519 (82.4)	358 (83.6)	161 (79.7)	0.140
History of antibiotic use in past 3 months	Yes	252 (40.0)	163 (38.1)	89 (44.1)	0.176
	No	378 (60.0)	265 (61.9)	113 (55.9)	0.138
Comorbidity	Yes	61 (9.6)	45 (10.5)	16 (7.9)	0.382
	No	569 (90.4)	383 (89.5)	186 (92.1)	0.162
Types of comorbidities	DM	15 (24.6)	13 (28.9)	2 (12.5)	0.313
	HTN	18 (29.5)	12 (26.7)	6 (37.5)	0.319
	HIV	7 (11.5)	6 (13.3)	1 (6.3)	0.423
	Others^d^	21 (34.4)	14 (31.1)	7 (43.7)	0.288

Abbreviations: AICU, adult intensive care unit; DM, diabetes mellitus; HIV, human immunodeficiency virus; HTN, hypertension; IQR, interquartile range; NA, not applicable; NAP-AMR, National Action Plan on Antimicrobial Resistance; NICU, neonatal intensive care unit; SCD, sickle cell disease; SSTI, skin and soft tissue infection. Bolded p-values: highlighting statistically significant results (p < 0.05).

a *P*-values calculated using a two-sample proportion test.

b During NAP-AMR: AICU (n = 29), neonatology unit (n = 10), NICU) (n = 5), and gynecology and obstetrics (n = 21). After NAP-AMR: AICU (n = 18), gynecology and obstetrics (n = 10), neonatology unit (n = 7), NICU (n = 3), and oncology (n = 2).

c During NAP-AMR: gentamicin (n = 38), erythromycin (n = 4), meropenem (n = 2), and azithromycin (n = 1). After NAP-AMR: gentamicin (n = 1).

d During NAP-AMR: HTN+DM (n = 6), SCD (n = 5), asthma (n = 2), and HTN+HIV (n = 1). After NAP-AMR: cancer (n = 6) and SCD (n = 1).

### *Prevalence of microbiologically confirmed SSTIs among patients with clinically diagnosed SSTIs*

**Figure 1:**
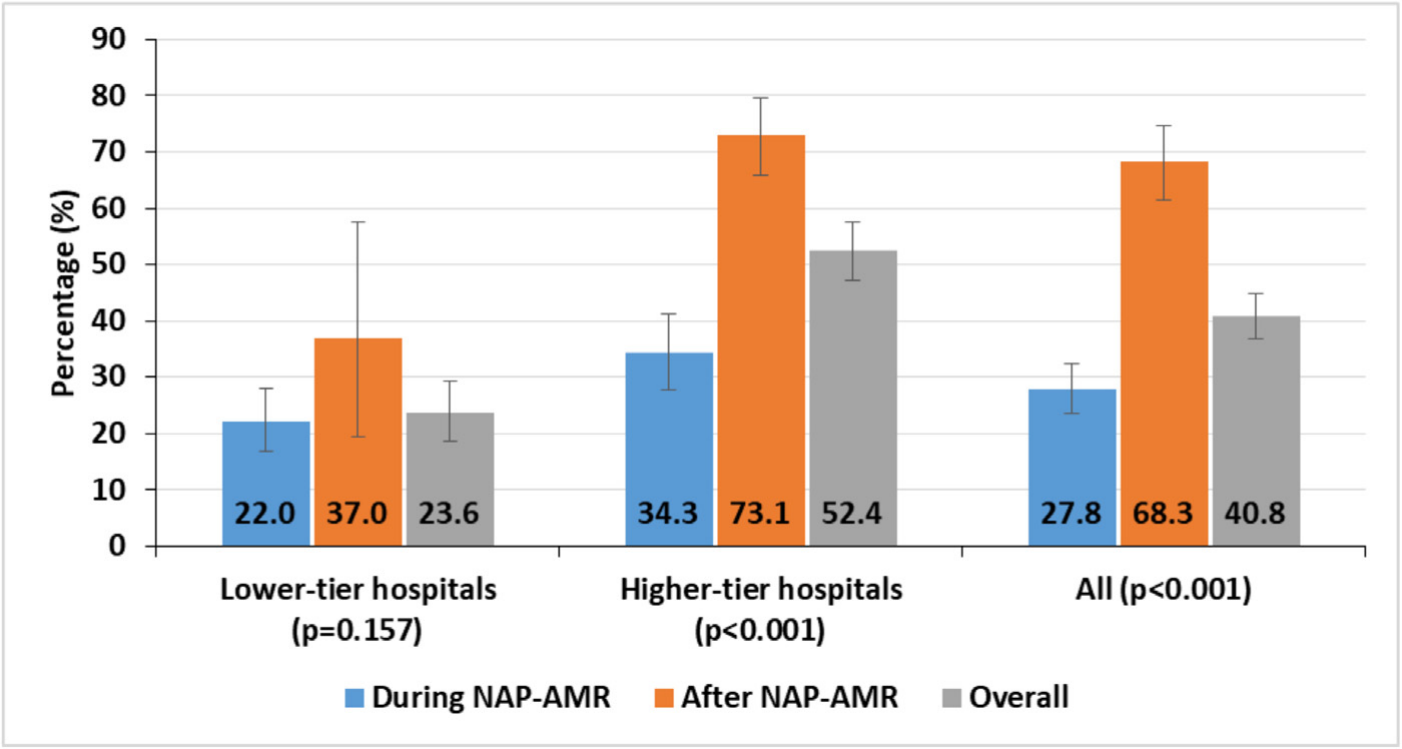
Prevalence of laboratory-confirmed SSTIs among patients with clinically diagnosed SSTIs. Error bars show 95% confidence intervals. P-values were obtained using the chi-square test.

Abbreviations: NAP-AMR, National Action Plan on Antimicrobial Resistance; SSTI, skin and soft tissue infection.

Of 630 patients, 257 had positive pus/pus swab cultures, yielding a 40.8% (95% CI: 36.9-44.7%) prevalence of microbiologically confirmed SSTIs. Prevalence increased significantly from 27.8% (119/428; 95% CI: 23.6-32.3%) during NAP-AMR to 68.3% (138/202; 95% CI: 61.4-74.7%) after NAP-AMR (*P* < 0.001). SSTIs were more common in higher-tier hospitals (52.4%, 197/376; 95% CI: 47.2-57.5%) than lower-tier hospitals (23.6%, 60/254; 95% CI: 18.5-29.3%; *P* < 0.001). In higher-tier hospitals, prevalence rose from 34.3% (69/201) to 73.1% (128/175; *P* < 0.001), whereas in lower-tier hospitals, it increased from 22.0% (50/227) to 37.0% (10/27; *P* = 0.157) (Figure 1).

### *Proportions of bacterial species causing SSTIs*

A total of 314 bacterial isolates were detected from 257 culture-positive samples, comprising 143 isolates from 119 positive cultures during NAP-AMR and 171 isolates from 138 positive cultures after NAP-AMR. Polymicrobial growth occurred in 19 samples during NAP-AMR (five with three and 14 with two bacterial species) and in 25 samples after NAP-AMR (eight with three and 17 with two bacterial species). Gram-negative bacteria (GNB) predominated in both periods, accounting for 91.6% (131/143) during NAP-AMR, of which 88.5% (116/131) were Enterobacterales, and 83.0% (142/171) afterward, with 66.2% (94/142) identified as Enterobacterales. During NAP-AMR, *K. pneumoniae* were most frequent (26.6%; n = 38), followed by *E. coli* (25.2%; n = 36) and *Proteus* spp. (15.4%; n = 22). After NAP-AMR, *E. coli* predominated (18.2%; n = 31), followed by *K. pneumoniae* (15.2%; n = 26) and *P. aeruginosa* (14.0%; n = 24). All second confirmatory samples collected from patients with initial growth of rare or uncommon bacterial species yielded concordant positive cultures, with *Aerococcus viridans, Delftia acidovorans, Acinetobacter pittii*, and *Proteus* spp. (*P. rettgeri, P. stuartii*, and *P. terrae*) isolated exclusively after NAP-AMR (Table 2). Notably, rare/uncommon bacterial species were isolated from patients with fever and prolonged hospitalization (≥3 months) at a zonal referral hospital.

**Table 2:** Proportions of bacterial species causing laboratory-confirmed SSTIs.

Gram-stain reaction	Bacterial species	During NAP-AMR (n = 143)	After NAP-AMR (n = 171)
		n (%)	n (%)
Gram-negative bacteria	*Klebsiella pneumoniae*	38 (26.6)	26 (15.2)
	*Escherichia coli*	36 (25.2)	31 (18.1)
	*Proteus* spp.	22 (15.4)	14 (8.2)
	*Enterobacter* spp.	13 (9.1)	10 (5.8)
	*Acinetobacter* spp.	6 (4.2)	23 (13.4)
	*Pseudomonas aeruginosa*	7 (4.9)	24 (14.0)
	*Serratia marcescens*	4 (2.7)	1 (0.6)
	*Klebsiella oxytoca*	2 (1.4)	1 (0.6)
	*Aeromonas hydrophila*	2 (1.4)	0 (0.0)
	*Citrobacter freundii*	1 (0.7)	2 (1.1)
	*Stenotrophomonas maltophilia*	0 (0.0)	4 (2.3)
	*Morganella morganii*	0 (0.0)	3 (1.7)
	*Citrobacter braakii*	0 (0.0)	2 (1.1)
	*Delftia acidovorans*	0 (0.0)	1 (0.6)
Gram-positive bacteria	*Staphylococcus aureus*	10 (7.0)	14 (8.2)
	*Enterococcus faecalis*	1 (0.7)	10 (5.8)
	*Staphylococcus epidermidis*	1 (0.7)	0 (0.0)
	*Enterococcus faecium*	0 (0.0)	2 (1.1)
	*Staphylococcus haemolyticus*	0 (0.0)	2 (1.1)
	*Aerococcus viridans*	0 (0.0)	1 (0.6)

Abbreviations: NAP-AMR, National Action Plan on Antimicrobial Resistance; SSTI, skin and soft tissue infection.

**During NAP-AMR:**

*Enterobacter* spp.: *E. cloacae* complex (n = 8), *E. hormaechei* (n = 4), and *E. aerogenes* (n = 1).

*Proteus* spp.: *P. mirabilis* (n = 15) and *P. vulgaris* (n = 7).

*Acinetobacter* spp.: *A. baumannii* (n = 4), *A. haemolyticus* (n = 1), and *A. nosocomialis* (n = 1).

**After NAP-AMR:**

*Enterobacter* spp.: *E. cloacae* complex (n = 5), *E. hormaechei* (n = 4), and *E. aerogenes* (n = 1).

Proteus spp.: P. mirabilis (n = 5), P. vulgaris (n = 4), P. rettgeri (n = 2), P. stuartii (n = 2), and P. terrae (n = 1).

*Acinetobacter* spp.: *A. baumannii* (n = 19), *A. haemolyticus* (n = 2), and *A. pittii* (n = 2).

### *Comparison of AMR percentages among bacterial pathogens causing SSTIs*

Overall, *Klebsiella* spp. demonstrated declining trends in AMR across all tested antimicrobial agents; however, the declines were not statistically significant (*P* > 0.05). Notably, no resistance to meropenem was detected during both study periods. In contrast, *E. coli* exhibited increasing AMR trends against most tested antimicrobial agents, with statistically significant increases observed for cephalosporins (cefpodoxime: 53.5% vs 83.8%, *P* = 0.018; cefotaxime: 46.4% vs 83.8%, *P* = 0.007; and cefuroxime: 60.7% vs 83.8%, *P* = 0.044). Although not statistically significant, reduced resistance among *E. coli* isolates was observed for gentamicin (32.1% vs 24.1%, *P* = 0.363) and tigecycline (7.1% vs 6.9%, *P* = 0.497). Additionally, emergence of meropenem resistance (0.0% vs 6.4%) was observed after NAP-AMR. Resistance to meropenem among other GNB rose from 2.6% to 21.0% (*P* = 0.327) owing to the increased isolation of *Acinetobacter* spp. and *P. aeruginosa* after NAP-AMR (Table 3).

**Table 3:** Comparison of antimicrobial resistance percentages among bacterial pathogens causing SSTIs.

Antibiotic agent	*Klebsiella* species			*Escherichia coli*			Other Gram-negative bacteria^a^		
	During NAP-AMR (n = 38-40)	After NAP-AMR (n = 26-27)	*P*- value^b^	During NAP-AMR (n = 36)	After NAP-AMR (n = 31)	*P*- value^b^	During NAP-AMR (n =9-54)	After NAP-AMR (n = 33-84)	*P*- value^b^
	% [95% CI]	% [95% CI]		% [95% CI]	% [95% CI]		% [95% CI]	% [95% CI]	
AMP	100 [90.5-100]	96.3 [81.0-99.9]	0.119	96.4 [81.6-99.9]	100 [88.8-100]	0.143	80.6 [62.5-92.5]	82.3 [65.5-93.2]	0.436
CAZ	70.3 [53.0-84.1]	62.9 [42.4-80.6]	0.306	28.6 [13.2-48.7]	51.6 [33.1-69.8]	0.142	12.5 [3.5-28.9]	31.6 [19.9-45.2]	0.221
CIP	43.2 [27.1-60.5]	42.3 [23.3-63.1]	0.481	50.0 [30.6-69.3]	72.4 [52.7-87.3]	0.089	20.5 [9.3-36.5]	54.0 [42.1-65.7]	0.042
CPD	91.9 [78.1-98.3]	81.5 [61.5-93.7]	0.122	53.5 [33.8-72.5]	83.8 [66.3-94.5]	0.018	38.7 [21.8-57.8]	51.5 [33.5-69.2]	0.248
CTX	91.9 [78.1-98.3]	81.5 [61.9-93.7]	0.122	46.4 [27.5-66.1]	83.8 [66.3-94.5]	0.007	19.3 [7.4-37.5]	44.7 [28.6-61.7]	0.135
CXM	91.9 [78.1-98.3]	81.5 [61.9-93.7]	0.122	60.7 [40.6-78.5]	83.8 [66.3-94.5]	0.044	22.2 [2.8 - 60.0]	41.6 [15.2-72.3]	0.314
GEN	89.2 [74.6-96.9]	74.1 [53.7-88.9]	0.075	32.1 [15.9-52.3]	24.1 [10.3-43.5]	0.363	21.6 [9.8-38.2]	46.6 [35.1-58.5]	0.098
SXT	94.6 [81.8-99.3]	84.6 [65.1-95.6]	0.102	89.3 [71.8-97.7]	89.7 [72.6-97.8]	0.481	36.1 [20.8-53.8]	69.1 [55.2-80.8]	0.018
TGC	NA	NA	NA	7.1 [0.9-23.5]	6.9 [0.8-22.8]	0.497	NA	NA	NA
TZP	72.9 [55.9-86.2]	62.9 [42.4-80.6]	0.242	17.8 [6.0-36.9]	48.3 [29.4-67.5]	0.116	18.9 [7.9-35.1]	50.7 [38.8-62.4]	0.060
MEM	0.0 [0.0-9.5]	0.0 [0.0-12.8]	NA	0.0 [0.0-12.3]	6.4 [0.7-21.4]	NA	2.6 [0.1-13.8]	21.0 [12.5-31.9]	0.327

Abbreviations: AMP, ampicillin; CAZ, ceftazidime; CI, confidence interval; CIP, ciprofloxacin; CPD, cefpodoxime; CTX, cefotaxime; CXM, cefuroxime; GEN, gentamicin; MEM, meropenem; NA, not applicable; NAP-AMR, National Action Plan on Antimicrobial Resistance; SSTI, skin and soft tissue infection; SXT, trimethoprim-sulfamethoxazole; TGC, tigecycline; TZP, piperacillin-tazobactam.

a Other Gram-negative bacteria included Enterobacterales and non-fermentative Gram-negative bacteria such as *Acinetobacter* spp. and *Pseudomonas aeruginosa*.

b *P*-values calculated using a two-sample proportion test.

For GPB, no significant change in resistance to trimethoprim-sulfamethoxazole, gentamicin, penicillin, erythromycin, levofloxacin, or tetracycline was observed after NAP-AMR. Moreover, we detected resistance to fosfomycin (13.3% vs 0.0%) in *Staphylococcus haemolyticus* (n = 2), rifampicin (6.7% vs 0.0%) in *S. aureus* (n = 1), and teicoplanin (3.8% vs 0.0%) in *S. haemolyticus* (n = 1) (Table 4).

**Table 4:** Trends of antibiotic resistance of Gram-positive bacteria causing SSTIs.

Antibiotic agent	Gram-positive bacteria		
	During NAP-AMR (n = 9-12)	After NAP-AMR (n = 12-29)	*P*-value^a^
	% [95% CI]	% [95% CI]	
CLI	0.0 [0.0-33.6]	0.0 [0.0-20.6]	NA
DAP	0.0 [0.0 33.6]	0.0 [0.0-20.6]	NA
ERY	55.5 [21.2-86.3]	62.5 [35.4-84.8]	0.397
FOS	0.0 [0.0-36.9]	13.3 [1.7-40.5]	NA
FUS	0.0 [0.0-36.9]	0.0 [0.0-21.8]	NA
GEN	22.2 [2.8-60.0]	50.0 [23.0-76.9]	0.242
LNZ	0.0 [0.0-30.8]	0.0 [0.0-13.2]	NA
LVX	40.0 [12.2-73.7]	38.5 [20.0-59.4]	0.521
TCY	33.3 [7.5-70.1]	30.8 [9.1-61.4]	0.472
MUP	0.0 [0.0-36.9]	0.0 [0.0-26.5]	NA
OXA	18.2 [2.3-51.8]	43.7 [19.7-70.1]	0.256
PEN	100 [63.1-100]	84.6 [54.5-98.1]	0.122
RIF	0.0 [0.0-33.6]	6.7 [0.2-31.9]	NA
SXT	87.5 [47.3-99.7]	93.3 [68.1-99.8]	0.327
TEC	0.0 [0.0-30.8]	3.8 [0.1-19.6]	NA
TGC	0.0 [0.0-30.8]	0.0 [0.0-15.4]	NA
VA	0.0 [0.0-30.8]	0.0 [0.0-13.2]	NA

Abbreviaitons: CI, confidence interval; CLI, clindamycin; DAP, daptomycin; ERY, erythromycin; FOS, fosfomycin; FUS, fusidic acid; GEN, gentamicin; LNZ, linezolid; LVX, levofloxacin; MUP, mupirocin; NA, not applicable; NAP-AMR, National Action Plan on Antimicrobial Resistance; OXA, oxacillin; PEN, penicillin; RIF, rifampicin; SSTI, skin and soft tissue infection; SXT, trimethoprim-sulfamethoxazole; TCY, tetracycline; TEC, teicoplanin; TGC, tigecycline; VA, vancomycin.

a *P*-values calculated using a two-sample proportion test.

### *Proportions and trends of WHO BPPs causing SSTIs*

**Figure 2:**
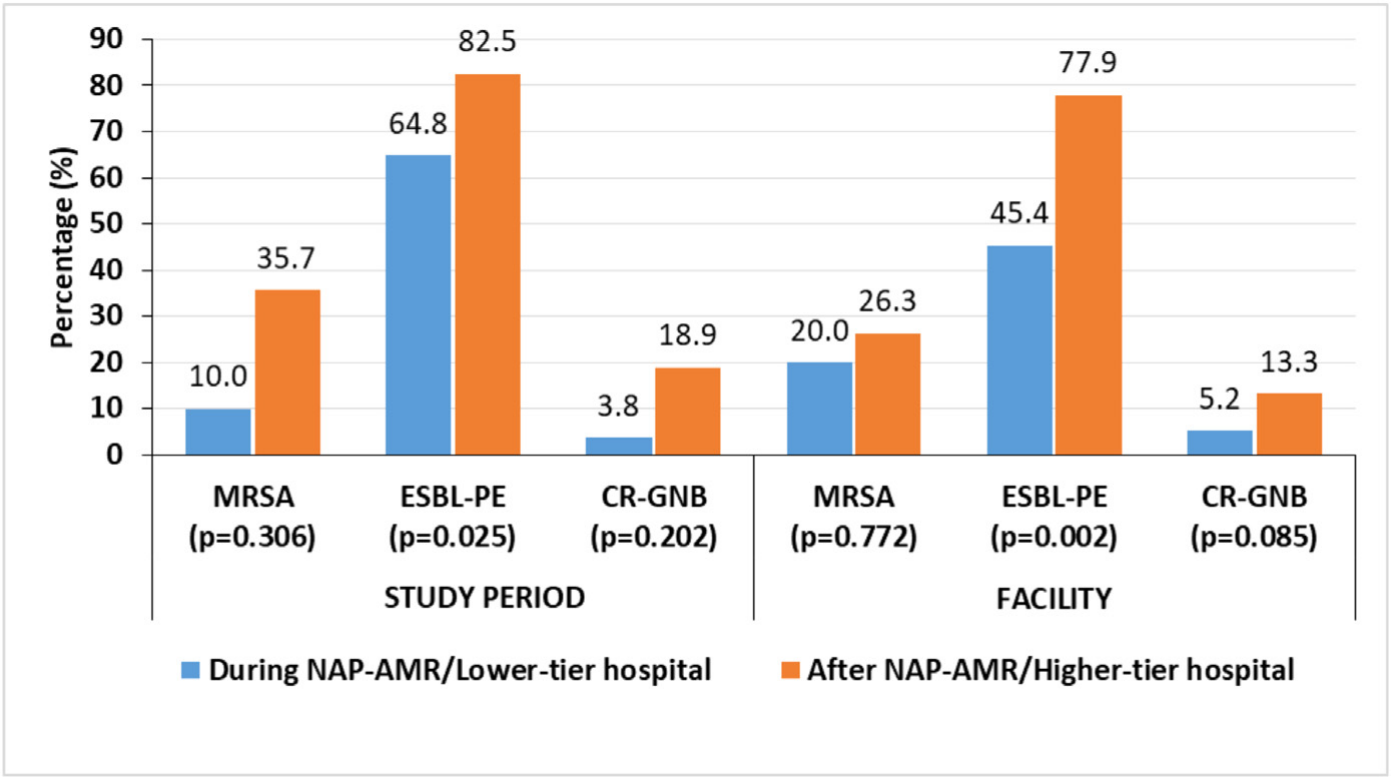
Proportions of WHO BPPs causing SSTIs by study period and level of health care facility. ESBL-PE comprised Escherichia coli and Klebsiella pneumoniae, and CR-GNB comprised all Enterobacterales and non-fermenting GNB with the exception of Stenotrophomonas maltophilia and Delftia acidovorans. P-values were obtained using the chi-square test.

Abbreviations: CR-GNB, carbapenem-resistant Gram-negative bacteria; ESBL-PE, extended-spectrum beta-lactamase-producing Enterobacterales; MRSA, methicillin-resistant Staphylococcus aureus; NAP-AMR, National Action Plan on Antimicrobial Resistance; SSTI, skin and soft tissue infection; WHO BPP, World Health Organization bacterial priority pathogen.

A total of 292 bacterial isolates were phenotypically tested: *S. aureus* (n = 24) for methicillin resistance; *E. coli* (n = 67) and *K. pneumoniae* (n = 64) for ESBL production; and Enterobacterales (n = 208), *A. baumannii* (n = 29), and *P. aeruginosa* (n = 31) for carbapenemase production. Overall, WHO BPPs (MRSA, ESBL-PE, and CR-GNB) accounted for 45.2% (132/292) of isolates, comprising 25.0% (6/24) MRSA, 72.5% (95/131) ESBL-PE, and 11.5% (31/268) CR-GNB. The prevalence of ESBL-PE increased significantly from 64.8% (48/74) to 82.5% (47/57) (*P* = 0.025). MRSA increased from 10.0% (1/10) to 35.7% (5/14, *P* = 0.306) and CR-GNB increased from 3.8% (5/131; *Proteus mirabilis* [n = 4] and *A. baumannii* [n = 1]) to 18.9% (26/137; *A. baumannii* [n = 15], *P. aeruginosa* [n = 4], *P. mirabilis* [n = 3], *Proteus vulgaris* [n = 2], *Enterobacter cloacae* complex [n = 1], and *Enterobacter hormaechei* [n = 1], *P* = 0.202). Higher-tier hospitals had more WHO BPPs, with ESBL-PE notably higher at 77.9% (85/109) compared with 45.4% (10/22) in lower-tier hospitals (*P* = 0.002) (Figure 2).

### *Factors associated with SSTIs caused by WHO BPPs*

The sociodemographic and clinical characteristics of the patients were analyzed to identify the factors associated with SSTIs due to WHO BPPs. Univariate logistic regression analysis (odds ratio: 3.72; 95% CI: 1.94-7.11; *P* < 0.001) and multivariable logistic regression analysis (odds ratio: 2.94; 95% CI: 1.42-6.07; *P* = 0.004) showed that patients in higher-tier hospitals had a significantly higher risk of SSTIs due to WHO BPPs than patients in lower-tier hospitals after adjusting for confounders (Table 5).

**Table 5:** Factors associated with skin and soft tissue infections by WHO bacterial priority pathogen.

Variables		Skin and soft tissue infections by WHO bacterial priority pathogen					
		Negative	Positive	Univariable regression analysis		Multivariable regression analysis	
		n = 160 (%)	n = 132 (%)	cOR [95% CI]	*P*-value	aOR [95% CI]	*P*-value
Median [IQR] age, years		27 [13-46]	29 [3.5-46]	-	0.449^a^		
Sex	**Female**	79 (54.1)	67 (45.9)	1			
	**Male**	81 (55.5)	65 (44.5)	0.95 [0.59-1.50]	0.814		
Residence	**Rural**	85 (56.7)	65 (43.3)	1			
	**Urban**	75 (52.8)	67 (47.2)	1.17 [0.74-1.85]	0.509		
Patient category	**Outpatient**	73 (62.9)	43 (37.1)	1		1	
	**Inpatient**	87 (49.4)	89 (50.6)	1.74 [1.07-2.80]	0.024	1.37 [0.81-2.30]	0.239
Facility level	**Lower-tier**	49 (77.8)	14 (22.2)	1		1	
	**Higher-tier**	111 (48.5)	118 (51.5)	3.72 [1.94-7.11]	<0.001	2.94 [1.42-6.07]	0.004
History of fever	**No**	86 (50.3)	85 (49.7)	1			
	**Yes**	74 (61.2)	47 (38.8)	0.64 [0.40-1.03]	0.067		
History of antibiotic use	**No**	92 (51.7)	86 (48.3)	1			
	**Yes**	68 (59.6)	46 (40.4)	0.72 [0.45-1.16]	0.183		
Currently receiving antibiotic	**No**	103 (49.8)	104 (50.2)	1		1	
	**Yes**	57 (67.1)	28 (32.9)	0.48 [0.28-0.82]	0.007	0.65 [0.32-1.31]	0.227
History of admission	**No**	129 (54.4)	108 (45.6)	1			
	**Yes**	31 (56.4)	24 (43.6)	0.92 [0.51-1.67]	0.795		
Chronic disease	**No**	146 (53.9)	125 (46.1)	1			
	**Yes**	14 (66.7)	7 (33.3)	0.58 [0.23-1.49]	0.261		
Study period	**During NAP-AMR**	87 (61.7)	54 (38.3)	1		1	
	**After NAP-AMR**	73 (48.3)	78 (51.7)	1.72 [1.08-2.74]	0.022	0.91 [0.48-1.69]	0.759

Abbreviations: aOR, adjusted odds ratio; CI, confidence interval; cOR, crude odds ratio; IQR, interquartile range; NAP-AMR, National Action Plan on Antimicrobial Resistance; WHO, World Health Organization.

a *P*-value calculated using a rank-sum (Wilcoxon) test.

## Discussion

This study revealed a marked increase in microbiologically confirmed SSTIs among patients with a clinical diagnosis of SSTI, especially in higher-tier hospitals, where the prevalence nearly doubled after the implementation of NAP-AMR. This indicates a connection between NAP-AMR and the increased detection of SSTIs, likely driven by enhanced diagnostic practices. Additionally, after NAP-AMR, the significantly higher proportion of patients enrolled before antibacterial exposure (likely reflecting antimicrobial stewardship advocacy following NAP-AMR) may have contributed to improved culture sensitivity, as previously reported [18,19].

Moreover, in line with previous studies in Mwanza [20,21] and Dar es Salaam [22], Tanzania, GNB were common pathogens implicated in SSTIs, with a predominance of *Klebsiella* spp., *E. coli*, and *Proteus* spp. during NAP-AMR. In contrast, *E. coli, Klebsiella* spp., and *P. aeruginosa* were predominant after NAP-AMR. Additionally, rare or uncommon bacterial species, including *A. viridans, D. acidovorans*, and *Proteus* variants (*P. rettgeri, P. stuartii*, and *P. terrae*), isolated exclusively after NAP-AMR, have previously been reported as opportunistic pathogens in immunocompromised patients or those with chronic conditions [23,24]. The exclusive detection of rare/uncommon bacterial species and the increased proportions of *P. aeruginosa* and *Enterococcus* spp. after NAP-AMR indicate an evolving microbial ecology associated with increased AMR [25]. This observation may also be attributed to improved diagnostic stewardship and enhanced laboratory capacity following interventions after NAP-AMR.

This study reported overall high AMR rates among bacterial pathogens causing SSTIs, consistent with a previous study conducted in Dar es Salaam, Tanzania [26]. However, AMR varied across bacterial species. *Klebsiella* spp. showed a decline in AMR, whereas *E. coli* showed a significant increase in AMR to cephalosporins, indicating a troubling trend for these widely used antibiotics. Surprisingly, although *K. pneumoniae* is generally considered intrinsically resistant to ampicillin owing to chromosomal SHV (Sulfhydryl Variable) beta-lactamase production, one *K. pneumoniae* isolate obtained after NAP-AMR in the present study was susceptible to ampicillin. A similar observation has previously been reported among clinical *K. pneumoniae* isolates in Italy [27], with differential expression or mutations of the *bla*_SHV_ gene being linked with such findings [28]. Concurrently, other GNB demonstrated significantly increased resistance to trimethoprim-sulfamethoxazole and ciprofloxacin, which are widely and commonly used antimicrobials in community and hospital settings [29], and are therefore associated with highly resistant bacterial pathogens. The increased AMR among other GNB may be attributable to the increased proportions of *Acinetobacter* spp. and *P. aeruginosa* after NAP-AMR, a finding consistent with a previous study on burn wound infections in hospitalized children [26]. Generally, the increasing levels of AMR in GNB, the primary causative agents of SSTIs in our setting, threaten the effectiveness of standard treatments. This is particularly concerning given their high resistance to 3GCs, which are widely used as surgical prophylaxis and for treatment of SSTIs (mostly surgical site infections) in Tanzania [10]. The increased AMR trends observed in this study may be attributable to increased antimicrobial use during the coronavirus disease 2019 pandemic, declared in Tanzania on March 16, 2020 [30]. Additionally, the higher proportion of patients from high-tier hospitals after NAP-AMR implementation may have contributed to the increased AMR trends. These facilities often manage severe and referred cases, which may have already been exposed to antimicrobials and frequently require prolonged hospitalization, broad-spectrum antimicrobial therapy, and multiple invasive procedures, all of which increase the risk of SSTIs caused by multidrug-resistant (MDR) pathogens [31]. Conversely, the lower sample volumes from lower-tier hospitals after NAP-AMR may reflect referral of SSTI cases to higher-tier hospitals for specialized management. This underscores the urgent need to update the Standard Treatment Guidelines and National Essential Medicines List for Tanzania Mainland and develop hospital level–specific antibiograms [10]. Moreover, routine epidemiological studies, especially AMR surveillance, are warranted to track emerging resistance patterns and guide empirical treatment, IPC practices, and stewardship programs.

Unlike GNB, AMR rates among GPB showed no significant changes. Of particular concern is the emergence of resistance to fosfomycin, rifampicin, and teicoplanin, even among a few isolates, as these drugs are rarely used in clinical settings [10]. Remarkably, *S. haemolyticus* was exclusively isolated after NAP-AMR, exhibiting resistance to fosfomycin and teicoplanin. Similar resistance patterns have previously been reported in *S. haemolyticus* strains associated with urinary tract infections in this setting [32]. This observation may indicate the onset of broader resistance or cross-infection with urinary tract infection isolates [32].

This study revealed a substantial proportion (45.2%) of WHO BPPs among clinical isolates causing SSTIs (particularly ESBL-PE), especially among patients in higher-tier hospitals, where broad-spectrum antimicrobials (particularly 3GCs) are used more frequently. Additionally, the study revealed an increase in the proportion of CR-GNB among *A. baumannii, P. aeruginosa, Proteus* spp., and *Enterobacter* spp., particularly in higher-tier hospitals. These findings are consistent with those of an earlier study [32]. The growing resistance to meropenem, a last-resort antibiotic for managing MDR infections, represents a significant public health challenge.

Furthermore, logistic regression analysis revealed a significant association between higher-tier hospitals and an increased risk of SSTIs caused by WHO BPPs, in line with a previous study [32]. Higher-tier hospitals often manage severe cases that require multiple invasive procedures, increased patient turnover and density, extensive use of broad-spectrum antimicrobials, and increased patient exposure to MDR bacterial pathogens [31]. These factors collectively heighten the risk of acquiring MDR infections, including those caused by WHO BPPs.

The interventions implemented after NAP-AMR, including promotion of rational antimicrobial prescribing, increased utilization of bacteriology laboratories, and refresher training on diagnostic stewardship and laboratory procedures, may have strengthened SSTI case identification and enhanced laboratory diagnostic capacity. This was reflected by the increased proportion of culture-positive cases, the exclusive detection of rare bacterial species, and the lower proportion of patients exposed to antimicrobials at enrollment after NAP-AMR compared with during NAP-AMR, consistent with findings from previous studies [33,34].

### *Study limitations*

The limitations of this study include the lack of anaerobic bacterial and fungal cultures. Although these organisms account for a relatively small proportion of SSTIs compared with aerobic bacteria, their exclusion may have led to underestimation of microbiologically confirmed SSTIs among clinically diagnosed patients. Additionally, comparison of findings before and after implementation of the NAP-AMR may have been affected by the smaller number of samples collected after NAP-AMR, particularly from lower-tier hospitals. Moreover, the study did not distinguish between hospital-acquired and community-acquired SSTIs, which may have limited the interpretation of AMR data across hospital and community settings.

## Conclusion

This study demonstrates important temporal changes in the epidemiology of SSTI pathogens following NAP-AMR implementation, characterized by a shift from *Klebsiella* spp. to *E. coli* as the predominant causative organism and a concerning rise in resistance to 3GCs. The high proportion of WHO BPPs underscores the substantial clinical and public health implications of AMR in SSTIs. Furthermore, the emergence of less common pathogens after NAP-AMR suggests evolving pathogen diversity that may complicate empirical treatment strategies. Collectively, these findings highlight the need for continuous, facility-level AMR surveillance, strengthened antimicrobial stewardship, and regular updating of treatment guidelines to ensure effective management of SSTIs.

## CRediT authorship contribution statement

**Vitus Silago:** Conceptualization, Data curation, Formal analysis, Funding acquisition, Methodology, Visualization, Writing – original draft, Writing – review & editing. **Katarina Oravcova:** Conceptualization, Funding acquisition, Methodology, Writing – review & editing. **Louise Matthews:** Conceptualization, Funding acquisition, Methodology, Writing – review & editing. **Stephen E. Mshana:** Conceptualization, Funding acquisition, Methodology, Writing – review & editing. **Heike Claus:** Conceptualization, Funding acquisition, Methodology, Supervision, Writing – review & editing. **Jeremiah Seni:** Conceptualization, Funding acquisition, Methodology, Supervision, Writing – review & editing.

## Declaration of competing interest

The authors have no competing interests to declare.

## Appendix A Supplementary materials

Supplementary Data S1 Supplementary Raw Research Data. This is open data under the CC BY license http://creativecommons.org/licenses/by/4.0/ Supplementary Data S1
